# Ageism and LGBTIQA+ Phobia: A Scoping Review of Barriers in Healthcare

**DOI:** 10.3390/healthcare14193276

**Published:** 2026-10-02

**Authors:** Andrea Paz-Lorenzo, Francisco Javier Castro-Molina, Jesús Manuel García-Acosta

**Affiliations:** 1 Canary Islands Health Service, 38071 Santa Cruz de Tenerife, Spain; alu0101485386@ull.edu.es (A.P.-L.); extjgarciaa@ull.edu.es (J.M.G.-A.); 2 Nuestra Señora de Candelaria School of Nursing, University of La Laguna, 38010 Santa Cruz de Tenerife, Spain; 3 Research Group on Care Throughout the Life Course–Health Management, Canary Islands Institute of Health Research (IISC), 38009 Santa Cruz de Tenerife, Spain

**Keywords:** healthcare disparities, nursing care, LGBTIQA+ phobia, sexual and gender minorities, older adults, ageism, barriers to healthcare access

## Abstract

Introduction: Population ageing and increasing recognition of sexual and gender diversity have highlighted health inequalities affecting older LGBTIQA+ people. Ageism and LGBTIQA+ phobia intersect, creating specific barriers to healthcare. Objective: To map and synthesize evidence on healthcare barriers affecting older LGBTIQA+ people and their consequences from a nursing perspective. Methods: A scoping review was conducted following PRISMA-ScR and Joanna Briggs Institute guidance. The review question was developed using the PCC framework. Searches were performed in PubMed, CINAHL, Scopus, and the Cochrane Library. Nineteen sources were included. Results: Structural barriers included inadequate professional training, cisheteronormativity, and limited inclusive policies. Interpersonal barriers comprised discriminatory attitudes, identity invisibility, misgendering, and fear of disclosure. Social barriers included stigma, isolation, and socioeconomic disadvantages. These barriers were associated with delayed care-seeking, reduced service utilization, and poorer physical, mental, and social health outcomes. Discussion: The findings indicate that inequalities cannot be explained by age or LGBTIQA+ identity alone, but by their interaction with socioeconomic and psychosocial vulnerabilities across the life course. Community networks may provide protection, although their availability is unequal. Nursing education, identity-affirming protocols, inclusive organizational policies, and accountability mechanisms are therefore required. Conclusions: Older LGBTIQA+ people experience cumulative barriers within healthcare and long-term care. Longitudinal and intervention research is needed to evaluate effective strategies for culturally safe and equitable care. The protocol was registered in the Open Science Framework on 8 July 2026.

## 1. Introduction

Population ageing is one of the major demographic and societal challenges facing contemporary societies, with a direct impact on healthcare systems and the organization of care [1,2].

The increase in life expectancy has led to a growing demand for healthcare services and has highlighted the need to develop policies and models of care that adequately address the needs of older adults [1,2,3]. In this context, systematic discrimination against individuals based on age—analogous to racism and sexism—known as ageism, a term coined by Robert Butler in 1968 [4], has gained increasing attention. Ageism is understood as the set of stereotypes, prejudices, and discriminatory practices directed at individuals because of their age, which may adversely affect their well-being, autonomy, and access to social and healthcare resources [5].

Ageism is not confined to the social sphere but also manifests within healthcare services, influencing how older adults are perceived and cared for by healthcare professionals. Recognizing the presence of ageism in healthcare settings is essential to promoting more equitable, person-centered care, particularly within disciplines such as nursing, which plays a pivotal role in the direct and continuous care of older adults [2,5,6].

In recent years, there has been growing academic interest in the living conditions and ageing experiences of older LGBTIQA+ people (Lesbian, Gay, Transgender, Bisexual, Intersex, Queer, Asexual, and other gender- and sexually diverse identities), bringing greater visibility to realities that remained overlooked for many years [7,8]. Nevertheless, studies focusing on the healthcare of older LGBTIQA+ people have documented experiences of discriminatory treatment, the invisibility of sexual orientation and gender identity, and care that is insufficiently responsive to diversity. These experiences contribute to mistrust of healthcare services and may result in reduced utilization of healthcare [7,8,9].

From the perspective of active ageing, as proposed by the World Health Organization (WHO), factors such as social participation, security, and equitable access to healthcare are fundamental to healthy ageing [1,3,9,10]. However, older LGBTIQA+ people continue to encounter barriers arising from the persistence of cisheteronormativity, discrimination based on sexual orientation and gender identity, and the lack of recognition of their specific needs. Phenomena such as returning to the closet in later life, fear of rejection, and the absence of adequate support networks have been identified as barriers that limit their equitable access to social and healthcare resources [7,11].

Within healthcare settings, these inequalities give rise to both structural and interpersonal barriers. Furthermore, fear of prejudicial attitudes among healthcare professionals has been shown to lead some older LGBTIQA+ people to conceal important aspects of their identity, thereby hindering the delivery of comprehensive, high-quality care [6,7,8,11].

In this context, the Minority Stress Model proposed by Meyer for sexual minorities, together with the extension developed by Testa et al. specifically for transgender and gender-diverse people, provides a relevant theoretical framework for understanding health inequalities among the LGBTIQA+ population [12,13]. These models propose that individuals belonging to sexual and gender minorities are exposed to specific stressors arising from their social position, in addition to the general stressors of everyday life. These stressors may be distal, such as experiences of discrimination or rejection, or proximal, such as fear of stigma or the internalization of negative attitudes towards one’s own identity. Together, these stressors may adversely affect both physical and mental health, as well as individuals’ relationships with healthcare services, influencing how they access and utilize healthcare [12,13,14,15].

From a nursing perspective, the literature highlights the importance of providing competent, inclusive, and non-discriminatory care, recognizing sexual and gender diversity as a social determinant of health. The lack of specific education and training on sexual diversity and ageing within nursing curricula and clinical practice contributes to the persistence of barriers in the therapeutic relationship between older LGBTIQA+ people and nursing professionals [6,11,16].

Evidence suggests that health inequalities among the LGBTIQA+ population are not attributable solely to age but also to structural disparities within the healthcare system compared with heterosexual and cisgender older adults [7,8,9,17]. In this regard, nursing plays a pivotal role in reducing health inequities and improving the quality of comprehensive care [11,16,17].

In the Spanish context, Law 4/2023 of 28 February, on the Real and Effective Equality of transgender People and the Protection of the Rights of LGBTI+ People, establishes an advanced legal framework that includes specific measures for older LGBTI+ people within the health and social care sectors, with the aim of ensuring safe, discrimination-free environments that are responsive to their needs [18]. Furthermore, Spain ranked first in ILGA-Europe’s Rainbow Map 2026 for the first time, achieving one of the highest scores in Europe for the strength of its legislation and public policies protecting the rights of LGBTI+ people [19].

Against this background, a comprehensive and systematic overview of the available evidence on the barriers affecting older LGBTIQA+ people within healthcare systems is needed. Accordingly, the aim of this scoping review was to map and synthesize the available scientific evidence on the barriers to healthcare access and healthcare provision affecting older LGBTIQA+ people, describing how ageism and LGBTIQA+ phobia have been conceptualized and examining their consequences for healthcare utilization and health outcomes from a nursing perspective.

In doing so, the review brings together both studies that explicitly address age-related discrimination in healthcare and sources that, while not labelling ageism as such, report barriers and inequalities affecting older LGBTIQA+ people that are consistent with ageist and cisheteronormative dynamics.

While recent reviews have examined discrimination in healthcare among older transgender individuals and the healthcare experiences of LGBTIQA+ older adults more broadly [20,21], the present scoping review extends this work by explicitly focusing on the intersection between ageism and LGBTIQA+ phobia, organizing barriers into structural, relational and social domains, and examining their implications for nursing practice and education.

## 2. Materials and Methods

Given the exploratory nature of the topic and the heterogeneity of the available evidence, a scoping review was considered the most appropriate approach for mapping the existing body of knowledge, identifying the types of available evidence, key concepts, and research gaps relating to ageism and LGBTIQA+ phobia among older adults.

This scoping review was conducted in accordance with the methodological framework developed by the Joanna Briggs Institute (JBI) and is reported following the Preferred Reporting Items for Systematic Reviews and Meta-Analyses Extension for Scoping Reviews (PRISMA-ScR) guidelines [22,23].

The study protocol was prospectively registered with the Open Science Framework (OSF) (Registration DOI: 10.17605/OSF.IO/52YJM). The completed PRISMA-ScR checklist [23] is provided as Supplementary File S1.

### 2.1. Study Design and Development of the Research Question

To guide the literature search and define the scope of the review, the Population–Concept–Context (PCC) framework recommended by the Joanna Briggs Institute (JBI) for scoping reviews was employed [24]. In this review, the Population (P) comprised LGBTIQA+ people aged 65 years and over; the Concept (C) encompassed ageism and LGBTIQA+ phobia, understood as barriers, forms of discrimination, and inequalities affecting access to and the provision of healthcare; and the Context (C) included a range of healthcare and social care settings, such as primary care, hospital care, mental health services, long-term care, and community-based services.

Based on this framework, the review question was formulated as follows: What is known from the scientific literature about the barriers to healthcare experienced by older LGBTIQA+ people, including ageism and LGBTIQA+ phobia, and what are the consequences of these barriers for access to healthcare services and health outcomes?

### 2.2. Search Strategy

A literature search was conducted between January and July 2026 in specialist health science databases to identify relevant sources of evidence on ageism and LGBTIQA+ phobia among older LGBTIQA+ people. The databases searched were PubMed, the Cumulative Index to Nursing and Allied Health Literature Complete (CINAHL), Scopus, and the Cochrane Library.

Search strategies were developed using controlled vocabulary from both DeCS (Health Sciences Descriptors) and MeSH (Medical Subject Headings), combined with the Boolean operators AND-OR to refine the search results. The search terms were organized according to the PCC framework. Population: older adults/ageism, elderly; sexual and gender minorities. Concept: ageism; barriers to healthcare access. Context: healthcare disparities; nursing care; health services.

Specific filters were applied according to the characteristics of each database, as detailed in the search strategy table (Table 1).

### 2.3. Selection of Sources of Evidence, Data Extraction, and Data Synthesis

Inclusion criteria were established to refine the search and select the most relevant sources of evidence:

- Studies primarily focused on older adults (≥65 years old) belonging to sexual and gender minorities.
- Publications addressing aspects related to nursing care or healthcare services.
- Studies addressing health inequalities, ageism, and/or LGBTIQA+ phobia affecting older LGBTIQA+ people.
- Quantitative, qualitative, and mixed-methods studies, previous reviews, and policy documents reporting empirical data on barriers to healthcare.
- Published in English or Spanish.
- Studies published in the last 5 years.

Although the PCC framework defined the Population as LGBTIQA+ people aged 65 years and over, we adopted a pragmatic life-course approach during screening. For the PubMed search, an adults ≥ 65 years filter was applied to identify studies explicitly focused on older adults, whereas no age filters were used in CINAHL, Scopus, or the Cochrane Library. We therefore included studies that either reported age-disaggregated findings for older participants or focused on adults in mid- to later life (typically ≥50 years), when barriers and health outcomes are directly relevant to the accumulation of disadvantage into older age. This heterogeneity in age ranges is acknowledged in the Limitations as a source of variability in how “older adult” is operationalized in the existing LGBTIQA+ health literature.

The following were excluded:

- Publications whose content was clearly irrelevant to the review question (e.g., those focusing on the general population without a specific analysis of older LGBTIQA+ people).
- Documents for which the full text could not be accessed despite reasonable attempts to obtain it.
- Commentaries and editorials that do not draw empirical evidence or clinical recommendations relevant to older LGBTIQA+ people.
- Studies related to dementia, ethics, and religion.

In line with the JBI recommendations for scoping reviews, methodological quality appraisal was not used as an exclusion criterion but rather as complementary information to describe the overall robustness of the body of evidence [24].

The selection process was conducted in two stages. In the first stage, an initial screening of the retrieved records was carried out by reviewing titles and abstracts to identify potentially relevant sources of evidence. In the second stage, the full-text articles selected were assessed in detail to determine whether they met the inclusion and exclusion criteria.

Two reviewers independently conducted the study selection and data extraction processes, recording information on the year of publication, country, methodological design, population characteristics, concepts related to ageism and LGBTIQA+ phobia, healthcare setting, and the main findings. No discrepancies requiring arbitration were identified.

Duplicate identification and removal were performed using Microsoft Excel^®^ (Version 2402; Microsoft Corp., Redmond, WA, USA). Records retrieved from the different databases were exported and merged into a single spreadsheet, where they were arranged alphabetically by title. Subsequently, the Remove Duplicates function was used to ensure accuracy and prevent the inclusion of duplicate studies.

Data were synthesized using a narrative and thematic approach, with the sources of evidence grouped according to predefined analytical categories. To facilitate understanding of the body of evidence and ensure alignment with the objectives of the scoping review, the extracted data were organized, classified, and summarized in a data extraction table. The information was synthesized into analytical categories consistent with the PCC framework and the JBI methodological recommendations for scoping reviews [22,24].

Table 2 presents this synthesis by organizing the sources of evidence according to the older LGBTIQA+ population studied, the conceptualization of ageism and LGBTIQA+ phobia, healthcare settings, study design, the different barriers identified (structural, interpersonal, and social), the consequences for access to and utilization of healthcare services and health outcomes, and the level and methodological quality of the available evidence.

### 2.4. Quality Assessment

In line with the primary objective of this scoping review, a formal critical appraisal of the methodological quality of the included studies was undertaken to describe the overall robustness of the identified literature.

In line with the primary objective of this scoping review, a formal critical appraisal of the methodological quality of the included studies was undertaken to describe the overall robustness of the identified literature. We followed the Joanna Briggs Institute (JBI) guidance for scoping reviews [24], which advises against excluding studies based on quality appraisal, and therefore used the appraisal results solely to contextualize the interpretation of the evidence.

For this purpose, we employed the Scottish Intercollegiate Guidelines Network (SIGN) [42] levels of evidence (Levels 1++ to 4 and Grades A to D), selecting this framework because it offers a concise, cross-design hierarchy that can be applied across analytical observational studies, qualitative research, mixed-methods designs, and reviews, rather than relying on multiple design-specific JBI checklists. Analytical observational studies (e.g., cross-sectional or cohort designs) were classified as level 2+ or 2− depending on risk of bias and analytical rigor; qualitative and other non-analytical designs (e.g., grounded theory, phenomenological studies, historical case studies) were assigned level 3; opinion papers were classified as level 4; and integrative or scoping reviews synthesizing observational and qualitative evidence were generally mapped to level 2−.

Table 3 presents the resulting level of evidence (LE) and grade of recommendation (GR) assigned to each study, which were used to describe the strength and consistency of the evidence rather than to determine eligibility.

### 2.5. Study Selection

The initial literature search identified a large number of records across the databases consulted (PubMed, CINAHL, Scopus, and the Cochrane Library), as detailed in Table 1, which presents the search strategies and applied limits. Following the removal of duplicate records and the screening of titles and abstracts, potentially relevant studies were selected for full-text assessment according to the predefined inclusion and exclusion criteria.

**Figure 1:**
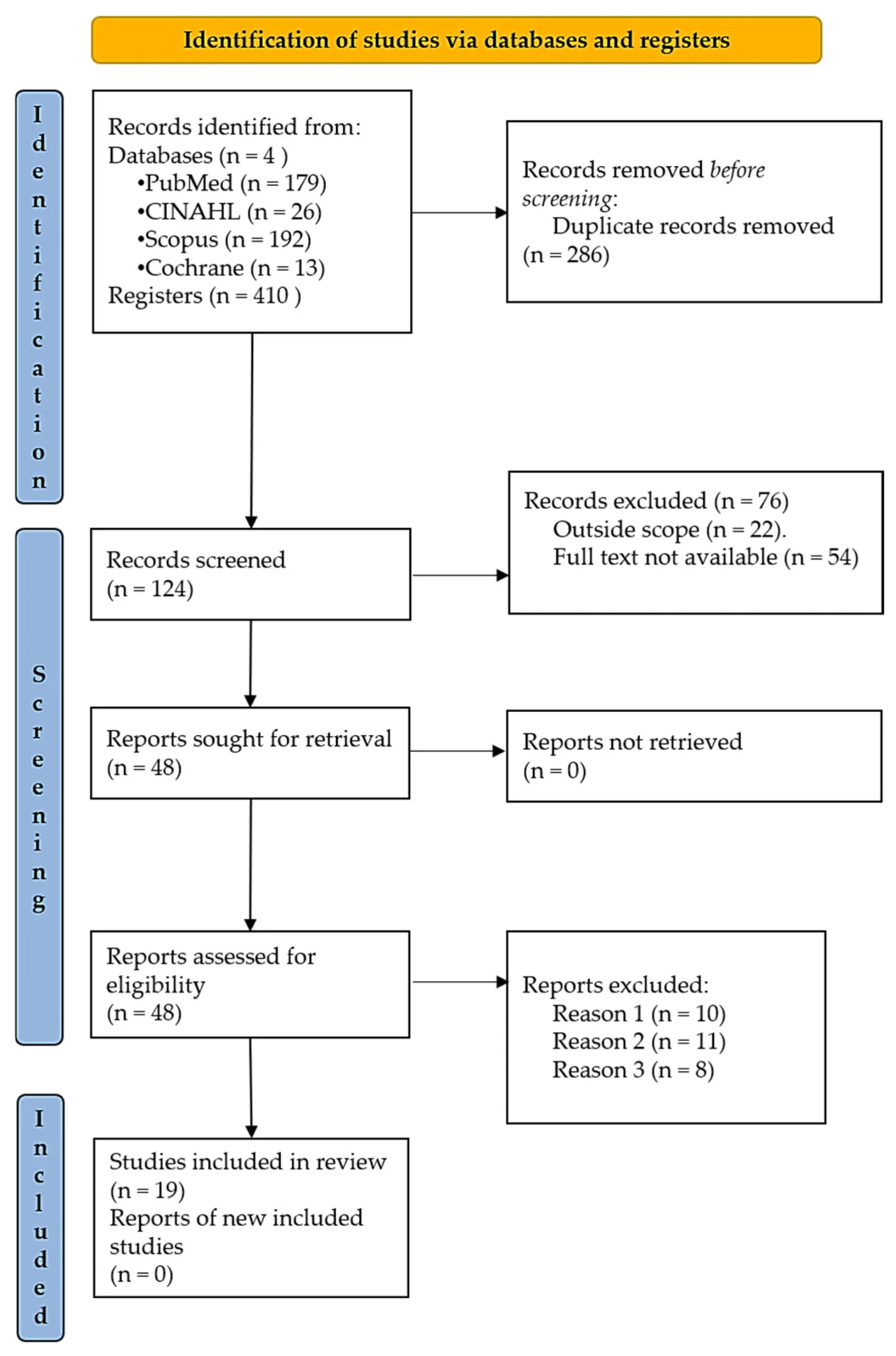
Adapted PRISMA 2020 Flow Diagram for Systematic Reviews which included Searches of Databases, Registers and Other Sources [43].

After the removal of 286 duplicate records, a total of 124 articles were screened based on their titles and abstracts. After title and abstract screening, the remaining full-text articles were assessed for eligibility. At this stage, 76 full-text articles were excluded: 22 because they were outside the scope of the review and 54 because the full text could not be obtained despite reasonable efforts. Forty-eight reports were retrieved and assessed for eligibility, of which 29 were excluded for the following reasons: population not aligned with the review question (Reason 1), lack of relevant data on the variables of interest (Reason 2), and ineligible publication type or study design (Reason 3). Ultimately, 19 sources of evidence were included, comprising quantitative, qualitative, and mixed-methods studies, as well as previous scoping reviews focusing on LGBTQIA+ older adults or on issues affecting their access to and experiences of healthcare. The processes of identification, screening, eligibility assessment, and inclusion are summarized in the PRISMA-ScR flow diagram (Figure 1).

## 3. Results

### 3.1. Characteristics of the Sources of Evidence

The included sources of evidence were conducted predominantly in high-income countries, including the United States [21,26,29,30,31,32,35,36,40], Taiwan [28], Brazil [25], Germany [34], the Netherlands [38], the United Kingdom [27], Australia [39,41], Israel [33], and New Zealand [37]. Together, they represent a wide range of healthcare and social care contexts, including primary care, hospital care, long-term care, community-based services, and population health surveys.

With regard to study design, the review included qualitative studies exploring the experiences of LGBTQIA+ older adults in residential aged care settings and healthcare professionals’ perspectives on culturally competent care [28,33,40,41], as well as a scoping review examining care for LGBTQIA+ older adults in residential aged care facilities [25]. Quantitative studies investigated health insurance coverage, access to healthcare, hospitalizations, and health behaviors [26,29,30,34,35,37,38]. Mixed-methods research examined psychosocial health among sexual and gender minority individuals with cancer [31], while comparative studies explored mental health and loneliness among older transgender adults receiving gender-affirming care [38]. The review also included scoping and integrative reviews addressing discrimination in healthcare experienced by older transgender individuals and the healthcare experiences of older adults with LGBTIQA+ identities [20,21,25].

The populations studied included LGBTQIA+ older adults living in long-term care facilities, older adults from sexual and gender minority groups receiving care across a range of healthcare settings, older transgender individuals attending gender identity clinics, and broader samples of sexual minority adults that provide valuable insights into life-course experiences and their implications for health and ageing in later life [26,27,32,35,36,38,40].

### 3.2. Narrative and Thematic Synthesis of the Identified Barriers

The narrative and thematic synthesis, organized according to the analytical categories presented in the synthesis table (Table 2), identified three main types of barriers: structural, relational, and social. It also highlighted their consequences for healthcare access and utilization, as well as their impact on health outcomes.

#### 3.2.1. Structural Barriers

Structural barriers were primarily related to the organization and design of healthcare and social care systems, professional training, and institutional policies. Several studies highlighted the limited provision of education on LGBTQIA+ health and ageing among nursing staff and other healthcare professionals, particularly in residential and long-term care settings [21,39]. They also identified the pervasive influence of cisheteronormativity within many healthcare services, where the specific needs of LGBTQIA+ older adults are not explicitly considered in the planning of resources and care protocols [25,28,41].

Furthermore, studies examining health insurance coverage and access to healthcare according to sexual orientation revealed persistent structural inequalities. Women from sexual minority groups were more likely to be uninsured, whereas insured men from sexual minority groups reported greater difficulty affording the healthcare they required. Other studies demonstrated the influence of socioeconomic disadvantage on patterns of hospital utilization and the demographic composition of LGBTQIA+ older adults accessing hospital services, highlighting the importance of economic and geographical factors [21,29,30,35].

#### 3.2.2. Relational Barriers

Relational barriers were associated with interactions between LGBTQIA+ older adults and healthcare professionals. Qualitative studies conducted in residential aged care facilities and other healthcare settings describe ambivalent or openly discriminatory attitudes, the invisibility of identities and relationships, misgendering, and fear of disclosing one’s identity because of anticipated rejection or mistreatment. Studies focusing on older transgender individuals reported discrimination in healthcare, including disrespectful treatment, the persistent use of non-affirmed names and gender markers, communication barriers, and breaches of confidentiality. These experiences frequently led individuals to avoid or delay seeking healthcare [20,21,25,28,39,41].

Other qualitative studies suggest that experiences within healthcare systems may be perceived as processes that fragment an individual’s sense of self. Achieving *wholeness* therefore requires intra-community support, self-determined care, and healthcare that affirms identity while addressing structural sources of harm [33,40]. These relational barriers have a direct impact on trust in healthcare services and on individuals’ willingness to seek care over time [28,33,40].

#### 3.2.3. Social Barriers

Social barriers refer to broader contextual factors, including stigma, social isolation, and socioeconomic disadvantages. Studies examining psychosocial health and real-world social networks indicate that LGBTIQA+ older adults experience a substantial burden of chronic stress resulting from social prejudice, family rejection, and limited recognition of their rights, which is associated with poorer emotional well-being and an increased risk of loneliness [26,31,33,34,35,36,38]. The evidence also shows that older transgender individuals report significantly higher levels of depressive symptoms, anxiety, and loneliness than their cisgender counterparts, largely explained by the cumulative effects of financial hardship, somatic problems, and social connectedness across the life course [26,38].

Socioeconomic disadvantages and geographical deprivation also influence patterns of hospital utilization, while distrust of formal services and the legacy of historical trauma undermine engagement with aged care services. As a result, many LGBTQIA+ older adults rely on informal support networks or seek to avoid institutional care whenever possible [32,35,41].

### 3.3. Consequences for Healthcare Access, Service Utilization, and Health Outcomes

Overall, structural, relational, and social barriers were consistently associated with delayed healthcare seeking, lower utilization of healthcare services, and poorer physical, mental, and social health outcomes among LGBTQIA+ older adults [20,21,28,30,31,35,36,41].

Several studies reported that inadequate professional training and the persistence of cisheteronormativity practices in residential aged care facilities and healthcare services lead LGBTQIA+ residents to conceal their identities, limit the expression of their relationships, and avoid using available services for fear of negative reactions, with detrimental effects on their well-being and mental health [25,31,32,36,38]. Structural inequalities in health insurance coverage and the ability to afford healthcare create practical barriers to accessing preventive services and treatment, particularly among women and men from sexual minority groups. In addition, less favorable health behavior patterns—including higher alcohol consumption, tobacco use, and poorer dietary habits—identified in population-based surveys suggest a greater accumulation of health risks that may manifest in later life [25,26,29,30,37,41].

With respect to mental health, the evidence indicates high levels of chronic stress, depressive symptoms, and loneliness among LGBTIQA+ older adults, particularly among older transgender individuals, whose real-world social networks do not always provide sufficient support. At the same time, several studies highlight the protective role of diverse community networks and collective empowerment processes in fostering resilience in the face of exclusion. These findings provide important insights for the development of interventions aimed at strengthening social support and improving access to culturally competent healthcare services [26,32,36,38,40].

### 3.4. Summary of the Evidence Map and Identified Gaps

Overall, the evidence map demonstrates a growing body of literature on ageism, LGBTQIA+ phobia, and health inequalities affecting LGBTQIA+ older adults. However, this evidence remains heavily concentrated in high-income countries and is dominated by descriptive and exploratory study designs.

Most studies focus on identifying barriers and associated health outcomes, whereas relatively few evaluate specific interventions aimed at reducing ageism and LGBTQIA+ phobia within healthcare and social care services, particularly in nursing practice and long-term care settings [21,25,28,29,35,37,38,40,41].

The review also revealed a limited number of studies explicitly led from a nursing perspective, together with a lack of evaluated protocols and educational programs that systematically integrate sexual and gender diversity into the care of older adults. Key knowledge gaps include the absence of longitudinal studies examining the cumulative impact of these barriers across the life course, the limited representation of primary care and community-based settings, and the scarcity of evidence on inclusive and culturally competent models of care that have been implemented and evaluated in terms of health outcomes and healthcare utilizations [21,25,28,39,41].

## 4. Discussion

The findings of this scoping review indicate that LGBTQIA+ older adults face a complex interplay of structural, relational, and social barriers in their interactions with healthcare and long-term care systems. These barriers are associated with delays in seeking healthcare, lower utilization of health services [25,28,29,30], poorer physical and mental health, and higher levels of loneliness, particularly among older transgender individuals and LGBTIQA+ older adults experiencing socioeconomic disadvantage [21,35,36,38].

The evidence map further demonstrates that inequalities in health insurance coverage, economic capacity, residential care organization, and social network composition are closely linked to experiences of discrimination, identity invisibility, and fear of rejection [21,25,26,29,30,31,32,33,34,35,36,37,39,40,41].

Together, these factors configure a pattern of cumulative vulnerability across the LGBTIQA+ life course that shapes healthcare access, service utilization, and health outcomes in later life [21,26,31,32,33,35,36,37,38,40,41].

### 4.1. Ageism, Active Ageing, and the Social Determinants of Health

The findings are consistent with previous evidence on ageism in healthcare services, which has shown that older adults frequently experience stereotyping, infantilizing attitudes, and exclusion from clinical decision-making. Among LGBTQIA+ older adults, these processes are further intensified by the cisheteronormative nature of many healthcare services and the absence of specific inclusive policies, thereby undermining the right to dignified care in later life as recognized in international human rights frameworks on the rights of older persons [2,6,7,9].

From the perspective of the World Health Organization’s Active Ageing framework, which advocates optimizing opportunities for health, participation, and security throughout the life course, the evidence included in this review suggests that these opportunities are not fully realized for LGBTQIA+ older adults [2,3]. Socioeconomic disadvantages, social isolation, fear of institutionalization in non-inclusive care settings, and the absence of adequate support networks act as key social determinants that limit the ability of LGBTQIA+ older adults to age actively, healthily, and successfully [7,11,21,26,35,38,41].

### 4.2. Minority Stress, Mental Health, and Intersectionality

The findings on mental health, real-world social networks, and loneliness among LGBTQIA+ older adults are consistent with Meyer’s Minority Stress Model, which links prejudice, expectations of rejection, identity concealment, and internalized stigma to an increased risk of psychological distress and mental health disorders [8,12,14]. Recent studies involving LGBTIQA+ older adults have shown that the cumulative effects of social stressors experienced throughout adulthood are associated with higher levels of depression and anxiety, whereas generativity and social participation function as psychosocial resources that can mitigate these adverse effects. The studies reviewed demonstrate that chronic stress and limited real-world social networks are associated with poorer emotional well-being among LGBTIQA+ older adults, highlighting the importance of perceived social support and diversity in the composition of real-world social networks [26,33,36,44,45].

Similarly, the evidence indicates that older transgender individuals experience significantly higher levels of depressive symptoms, anxiety, and loneliness than their cisgender counterparts, largely explained by the cumulative effects of financial hardship, somatic problems, and reduced social connectedness across the life course [38]. When examining the experiences of older transgender individuals, it is important to consider the role of cisnormativity in healthcare and the need for pluralistic theoretical frameworks capable of capturing the specificity of their lived experiences. This perspective reinforces the view that inequalities cannot be explained solely by older age or transgender identity, but rather by the intersection of these characteristics with other axes of vulnerability [14,38,46].

This perspective is consistent with intersectionality frameworks applied to LGBTQIA+ populations, which argue that systems of oppression intersect to produce distinct forms of inequality affecting women and sexual and gender minority groups that cannot be understood through isolated social categories [13,15]. The findings support an intersectional interpretation of health inequalities in LGBTQIA+ older adults by demonstrating how ageism, LGBTQIA+ phobia, socioeconomic disadvantage, and other forms of discrimination interact to generate barriers to healthcare access and utilization [13,15,21,25,35,41].

### 4.3. Implications for Nursing and Clinical Practice

The evidence confirms that the lack of specific education and training in sexual and gender diversity among nurses and other healthcare professionals constitutes a major barrier to the provision of culturally competent, inclusive, and non-discriminatory care [11,21,25,28,41]. Studies have shown that the quality of comprehensive care provided to LGBTQIA+ populations depends largely on the cultural competence of nursing teams and the explicit integration of sexual and gender diversity into clinical protocols and models of care. Qualitative research exploring nurses’ knowledge and experiences in caring for transgender individuals has identified substantial gaps in knowledge, attitudes, and perceived confidence, which contribute to experiences of inequity and mistreatment within healthcare services [11,16,20,25,28,41].

In this context, Madeleine Leininger’s Theory of Culture Care Diversity and Universality, together with its applications to sexual and gender diversity, provides a valuable framework for designing culturally competent nursing interventions that emphasize individualized care tailored to the specific needs of LGBTQIA+ people [47]. Accordingly, the findings of this evidence mapping support the systematic integration of sexual and gender diversity into undergraduate and postgraduate nursing curricula, as well as the development of mandatory continuing education programs for professionals working in residential aged care facilities, hospitals, and primary care settings. These programs should include content on ageism, LGBTQIA+ phobia, historical trauma, and strategies for delivering identity-affirming, culturally competent care [11,16,21,25,28,41].

### 4.4. Limitations of the Evidence Base and the Present Scoping Review

The identified body of evidence presents several limitations that should be considered when interpreting the findings. Most of the included studies were conducted in high-income countries and predominantly urban settings, limiting the generalizability of the findings to rural contexts and to healthcare systems with less developed policies on LGBTQIA+ sexual and gender diversity.

Descriptive and cross-sectional study designs predominated, whereas longitudinal studies examining the cumulative impact of these barriers across the life course were scarce. Likewise, there was a lack of rigorous evaluations of specific interventions aimed at reducing ageism and LGBTQIA+ phobia within healthcare and social care services.

It is important to note that relatively few of the included studies explicitly operationalize ‘ageism’ as a measured construct. Instead, most sources document barriers, discriminatory experiences and health inequalities affecting older LGBTIQA+ people, which we interpret through an ageism and LGBTIQA+ phobia lens when they reflect age-related and cisheteronormative exclusion in healthcare settings.

Regarding the review process, the literature search was limited to selected databases and predefined time periods, which may have resulted in the omission of relevant studies available in other repositories or published in languages other than those included in the search strategy. Consequently, pertinent studies indexed in interdisciplinary databases and published in other languages may have been overlooked. In addition, as no structured search of the grey literature was undertaken, relevant publications may have been missed, potentially introducing publication or reporting bias by limiting the comprehensiveness of the evidence map. Although an exploratory methodological quality appraisal was conducted using tools such as the Scottish Intercollegiate Guidelines Network (SIGN) criteria [42], this assessment was used solely to contextualize the interpretation of the evidence map and did not serve as a basis for excluding studies, in accordance with the recommendations of the Joanna Briggs Institute (JBI) for scoping reviews [22,24]. Finally, there was considerable heterogeneity in both the age ranges and the definitions used for “older adult” across the included sources. Some studies focused explicitly on participants aged 65 years and over, whereas others examined broader age bands (e.g., ≥50 years or general adult samples with age-stratified analyses). Together with variability in the LGBTQIA+ identity categories considered, this heterogeneity limits direct comparisons between findings and should be considered when interpreting the evidence. In addition, explicit operationalization of ageism was limited across the included literature. Our intersectional framing of ageism and LGBTIQA+ phobia therefore relies on conceptual alignment between the reported barriers and age-related and cisheteronormative dynamics, rather than on uniform measurement of ageism in all studies.

### 4.5. Implications for Research, Clinical Practice, and Health Policy

In light of these findings, several priorities for future research can be identified. Longitudinal studies are needed to examine the cumulative impact of ageism and LGBTQIA+ phobia on health outcomes and healthcare utilization among LGBTQIA+ older adults. In addition, rigorous evaluations of interventions specifically designed to reduce these barriers in residential aged care facilities, hospitals, and primary care settings are required [2,9,12,13].

Particular emphasis should be placed on developing nursing-led research that integrates theoretical frameworks such as Minority Stress Theory, intersectionality, and Madeleine Leininger’s Theory of Culture Care Diversity and Universality in order to generate evidence-based models of care tailored to the specific needs of LGBTQIA+ older adults [6,11,12,13,15,16,47].

From a clinical practice perspective, the findings support the implementation of identity-affirming care protocols, mandatory education and training programs, and robust monitoring and accountability mechanisms to ensure culturally safe care for LGBTQIA+ older adults [1,4,6,7,21,41]. From a health policy perspective, international frameworks on active ageing, the social determinants of health, and the human rights of older adults and LGBTQIA+ people provide a strong foundation for advancing legal and organizational reforms aimed at reducing the inequalities identified in this review. Integrating these perspectives into the planning of services for older adults and broader public health strategies is essential to advancing healthcare systems that recognize diversity and ensure dignified, safe, and equitable ageing for LGBTQIA+ older adults [3,5,11,25,28,35,41].

The landmark achievement of Spain ranking first in the ILGA-Europe Rainbow Map 2026, together with the enactment of Law 4/2023, reflects a favorable political and legal context for the protection of LGBTI+ rights. Nevertheless, the persistence of the barriers identified in this review suggests that the current challenge lies not in the absence of legislation, but in the effective implementation of inclusive policies across healthcare and long-term care services, as well as in the transformation of professional practice [18,19].

## 5. Conclusions

This scoping review demonstrates that ageism and LGBTQIA+ phobia intersect within healthcare and long-term care systems, giving rise to multiple structural, relational, and social barriers that adversely affect healthcare access, service utilization, and health outcomes among LGBTQIA+ older adults. These barriers are manifested through inadequate professional education and training in sexual and gender diversity, the persistence of cisheteronormativity within the organization of healthcare and long-term care services, socioeconomic inequalities, and insufficient social support networks, together creating a pattern of cumulative vulnerability across the life course.

The findings are consistent with the World Health Organization’s Active Ageing framework and the social determinants of health approach, demonstrating that the conditions required for healthy, participatory, and secure ageing are not fully realized for LGBTQIA+ older adults because of the combined effects of ageism, discrimination based on sexual orientation and gender identity, and structural socioeconomic disadvantage. The findings also reinforce the contributions of Minority Stress Theory and intersectionality by demonstrating that health inequalities affecting LGBTQIA+ older adults cannot be understood solely in terms of age or LGBTQIA+ identity, but rather through the interaction of multiple, intersecting systems of oppression.

From a nursing perspective, the evidence highlights the urgent need to integrate sexual and gender diversity into undergraduate and continuing professional education, to develop culturally competent and identity-affirming care protocols, and to establish robust governance and accountability mechanisms that ensure culturally safe care for LGBTQIA+ older adults.

The existence of a favorable legal and policy framework does not necessarily translate into the absence of barriers in everyday clinical practice. This underscores the need to examine how equality is implemented in practice within healthcare and long-term care services for LGBTQIA+ older adults.

The findings identify several priorities for future research, including the design and evaluation of targeted interventions to reduce ageism and LGBTQIA+ phobia, the development of longitudinal studies examining the cumulative impact of these barriers across the life course, and the expansion of nursing-led research across diverse healthcare and social care settings.

## Figures and Tables

**Table 1:** Search strategy and sources of evidence identified.

Database	Search Strategy	Specific Filters	Number of Articles Identified
PubMed	(Ageism) AND (sexual and gender minorities) AND (Healthcare Disparities) OR (barriers to access of health services)	Adults ≥ 65 years old 5 years Humans Spanish English Complete text	121
	(Ageism) AND (sexual and gender minorities) AND (nursing care).		5
	(Elderly) AND (sexual and gender minorities) AND (nursing care) AND (health services).		48
	(Ageism) AND (sexual and gender minorities) AND (nursing care).		5
CINAHL	(Ageism) AND (sexual and gender minorities) AND (Healthcare Disparities) OR (barriers to access of health services)	5 years Complete text Open access Electronic journal English	26
Scopus	(Elderly) AND (sexual and gender minorities) AND (nursing care) AND (health services) OR (barriers to access of health services)	5 years All open access English Nursing Humans-Aged Article	41
	(Ageism) AND (sexual and gender minorities) AND (nursing care).		31
	(Elderly) AND (sexual and gender minorities) AND (nursing care) AND (health services).		117
	(Ageism) AND (sexual and gender minorities) AND (nursing care).		3
Cochrane	(Ageism) AND (sexual and gender minorities) AND (Healthcare Disparities) OR (barriers to access of health services)	Last 5 years No filters	13

**Table 2:** Identified included studies by chart review.

First Author/Year	Context and Design	Population	Main Barriers	Consequences
Silva JR/2021 [25]	Brazil Qualitative study conducted in residential care homes for older adults.	Older LGBTI+ people living in long-term care facilities and nursing professionals/other care staff.	Lack of knowledge and training in LGBTI+ health. Ambivalent or negative attitudes among healthcare professionals towards LGBTI+ residents. Invisibility of LGBTI+ identities and relationships (e.g., same-sex couples).	Fear of disclosing one’s identity and adapting behaviors to “blend in”. Limitations in providing truly individualized and respectful care, with the potential to adversely affect well-being and mental health.
Kittle KR/2022 [26]	USA Cross-sectional quantitative study based on secondary analysis of study data.	LGBT adults aged 50–95 years; subgroup analyses were conducted according to identity (lesbian, gay, bisexual, and transgender), age, and educational level (*n* = 2536).	Differences in social network size, perceived social support, and sense of community belonging according to LGBT identity, age, and educational level; lower levels of social support and community belonging among bisexual men and some transgender people, reflecting inequalities in access to key social resources.	Lower levels of social support and community belonging among certain subgroups (e.g., bisexual men and some transgender people) were associated with greater psychosocial vulnerability and a potential risk of poorer health outcomes, highlighting that larger real-world social networks do not necessarily guarantee adequate support.
Matthews AK/2022 [27]	United Kingdom Opinion paper discussing ways to improve care for older LGBT+ people, based on a synthesis of previous evidence and clinical recommendations.	Healthcare professionals and services providing care for older LGBT+ people (not a direct empirical study, but a discussion of clinical practice and service organization).	Persistent barriers to healthcare were identified, including heteronormative assumptions, the lack of systematic collection of identity-related data, the invisibility of specific needs, and inadequate staff training.	It proposes organizational and educational changes to achieve care that is “equal but different”, recognizing the specific needs of older LGBT+ people. Failure to implement these changes perpetuates inequalities in access to healthcare, perceived quality of care, and trust in healthcare services.
Wang Y/2022 [28]	Taiwan Qualitative study based on interviews, applying the social ecological model.	Healthcare professionals provide care to older people from sexual and gender minorities (in residential care homes and other healthcare settings).	Intrapersonal: attitudes towards older LGBTIQA+ people, and limited knowledge and experience in providing their care. Interpersonal: concerns about interactions between LGBTIQA+ residents, cisgender heterosexual residents, and their families. Community: lack of resources and training programs, and limited support from managers and healthcare organizations. Society: an unfavorable social and political environment, and the absence of clear policies.	These barriers limit healthcare providers’ willingness and ability to deliver culturally competent care, thereby contributing to inequalities in quality of life and health among older LGBTIQA+ people.
Gavulic K/2024 [29]	USA Cross-sectional quantitative study involving almost one million participants aged 51–79 years from the Behavioral Risk Factor Surveillance System (BRFSS).	Older adults, comparing sexual and gender minorities with heterosexual and cisgender populations.	Before the age of 65 years, LGB+ individuals experienced greater disparities in health insurance coverage, access to healthcare, and health status compared with the heterosexual population. State-level disparities were identified, particularly in the southern and central regions of the United States.	Eligibility for Medicare improves health insurance coverage and access to healthcare for all individuals; however, it does not consistently reduce disparities in insurance coverage and healthcare utilization between sexual minorities and the heterosexual population.
Nguyen K/2024 [30]	USA Cross-sectional study using Behavioral Risk Factor Surveillance System (BRFSS) data from adults aged 18–64 years, comparing groups according to sexual orientation.	Adults from sexual minority groups (primarily lesbian, gay, and bisexual individuals). Although the study does not focus exclusively on older adults, it provides relevant evidence on access to healthcare in the context of a pandemic.	Sexual minority women were more likely to be uninsured than heterosexual women. Among sexual minority men, even those with health insurance experienced greater difficulty affording the healthcare they needed.	Lack of health insurance exacerbates disparities in healthcare access among sexual minority women, including reduced healthcare utilization and delays in receiving necessary care. Among insured sexual minority men, financial barriers to accessing healthcare persisted, indicating that health insurance alone does not eliminate disparities in access to care.
Franco-Rocha O/2025 [31]	USA Concurrent mixed-methods study (*n* = 295) combining quantitative and qualitative analyses of sexual and gender minority (SGM) respondents with gastrointestinal (GI) cancer.	Sexual and gender minority individuals with anal or colorectal cancer.	Clinical and demographic characteristics influenced mental health outcomes. Interactions with healthcare professionals, comorbidities, and treatment-related effects affected psychosocial well-being. The findings highlighted the need for culturally sensitive care that acknowledges historical trauma.	Poorer mental health than their cisgender heterosexual counterparts, with negative effects on satisfaction with care, social support, and access to mental healthcare resources. The authors recommended prioritizing peer support interventions and self-esteem promotion, alongside training healthcare professionals in culturally competent care.
Perone A/2024 [32]	USA Multidisciplinary historical case study combining community interviews and archival data to examine the exclusion of and resistance among transgender individuals.	Older transgender individuals, examined through the history of exclusion and empowerment within social and community services.	Longstanding history of exclusion of transgender individuals from social and healthcare services, alongside criminalization and institutional violence. Lack of dedicated services and failure to recognize the specific needs of transgender individuals within support systems.	Social exclusion created the conditions for collective empowerment through the development of self-efficacy, critical consciousness, peer connectedness, skill acquisition, and collective action to address social, economic, and political barriers. The findings provide important implications for social work and gerontological practice by highlighting the need to integrate historical memory and empowerment into care for older transgender individuals.
Sperling D/2024 [33]	Israel Interpretive qualitative study based on in-depth interviews, exploring the needs, experiences, and future expectations of older LGBTQ individuals.	Older LGBTQ individuals, who often lived alone, had limited support from their biological families, and relied heavily on community networks and chosen families.	Limited family support and a lack of formal services tailored to their needs. Fear of institutionalization in non-inclusive care settings. Economic barriers. Low levels of trust in mainstream ageing services.	High levels of concern about the future, increasing need to rely on community networks, and complex planning for later life. Increased risk of social isolation and inadequate support if healthcare and care services fail to adapt to the needs of diverse older populations.
Buczak-Stec E/2025 [34]	Germany Cross-sectional study using an online survey of adults aged 40–70 years (n ≈ 4000), including lesbian, gay, and bisexual individuals, as well as cisgender and transgender participants.	Middle-aged and older adults, comparing lesbian, gay, and bisexual individuals with heterosexual individuals in terms of preferences for long-term care and home care.	LGB individuals placed greater importance on urban location, allowing intimate relationships within residential care facilities, and the availability of LGBT-friendly or LGBT-exclusive care facilities. They also showed a lower preference for residential care facilities intended exclusively for heterosexual residents.	These differences in preferences reflect concerns about safety, acceptance, and recognition within long-term care settings. The findings highlight the need to design residential and home care services that provide inclusive environments, respect intimate relationships and non-traditional family structures, and offer care options specifically tailored to the needs of LGB communities.
May J/2025 [35]	USA Secondary analysis of clinical records (2018–2022) from a large healthcare system.	270 hospital admissions involving LGBTQ+ older adults (aged ≥50 years), classified according to the area deprivation index of their place of residence.	Unequal distribution of hospital admissions according to the level of socioeconomic disadvantage (low, moderate, and high). A higher proportion of individuals identifying as asexual and bisexual lived in areas of moderate or high socioeconomic disadvantage, suggesting distinct profiles of vulnerability within these groups.	Socioeconomic disadvantages were associated with differential patterns of hospital utilization and identity composition. The findings underscore the need to understand how poverty and socioeconomic deprivation shape healthcare utilization among LGBTQ+ older adults.
Constantinou C/2025 [20]	Scoping review based on a search of multiple databases (Scopus, PubMed, CINAHL, MEDLINE, ProQuest, among others) covering the previous 20 years.	Transgender individuals aged ≥65 years old across all healthcare settings; the review mapped studies examining discrimination in healthcare.	Anticipated and/or experienced discrimination by healthcare providers. Avoidance of healthcare services due to fear of mistreatment, misgendering, and forced disclosure of gender identity. Incongruence between official documentation (legal name and registered sex) and an individual’s chosen name and gender identity, creating barriers to healthcare access and continuity of care. Lack of competence and specific training among healthcare professionals, together with financial barriers.	Avoidance and delayed utilization of healthcare services, resulting in adverse effects on both physical and mental health. Victimization and cumulative vulnerability arising from the intersection of older age, transgender identity, and socioeconomic marginalization. The review concluded that policy reforms, inclusive clinical practices, and community-based support are essential to ensure safe and gender-affirming care for older transgender individuals.
Prasad A/2025 [36]	USA Mediation and moderation analysis using data from the national Aging with Pride: National Health, Aging, and Sexuality/Gender Study (NHAS), including LGBT adults aged 50 years and older.	LGBT older adults from across the United States; the study examined the size and composition of real-world social networks, including LGBT vs. non-LGBT members, as well as older vs. younger individuals.	Chronic stress was associated with poorer emotional well-being (including depression and loneliness), with this relationship being mediated by reduced perceived social support.	Larger real-world social networks, particularly those including other LGBT and older individuals, acted as moderators, buffering the effects of chronic stress on depressive symptoms and loneliness. These findings suggest that both the size and composition of real-world social networks are key considerations when designing support interventions for LGBT older adults.
Adams J/2025 [37]	New Zealand Secondary analysis of six waves of the New Zealand Health Survey.	67,426 participants aged ≥15 years old, including 1224 women and 703 men from sexual minority groups (lesbian, gay, and bisexual). Although not restricted to older adults, the study provides important evidence on behavioral health inequalities across the life course.	Sexual minority individuals exhibited poorer health behavior profiles than their heterosexual counterparts, including: Higher levels of alcohol consumption. Higher prevalence of tobacco use. Greater use of illicit drugs. Lower adherence to recommendations for fruit and vegetable consumption.	The overall pattern reveals behavioral health inequalities that accumulate over time and may contribute to a greater burden of disease in later life. The findings identify opportunities for targeted health promotion interventions aimed at sexual minority populations and emphasize the ethical responsibility of public health agencies to address these persistent health inequalities.
van Heesewijk J/2025 [38]	The Netherlands Comparative cross-sectional study conducted in a gender identity clinic and the general population.	72 transgender women and 39 transgender men aged 56–84 years, all of whom had received gender-affirming hormone therapy for at least 10 years, were compared in a 1:3 ratio with cisgender women and cisgender men.	Transgender women and men reported significantly higher levels of depressive symptoms, anxiety, and loneliness than their cisgender counterparts. These differences were largely explained by financial, psychological, somatic, and social factors, including the absence of a partner and lower health-related quality of life.	The cumulative effects of socioeconomic and psychosocial inequalities across the life course have a detrimental impact on mental health and loneliness in later life among transgender individuals, even after prolonged access to gender-affirming healthcare.
Scott K/2025 [39]	Australia Cross-sectional quantitative study using an online survey to examine concerns about ageing and access to aged care services.	LGBT+ adults and older adults living in Australia, with a particular focus on individuals anticipating a future need for long-term care services.	Concerns about discrimination and a lack of safety in residential aged care facilities and other aged care services. Perceived lack of staff training in LGBTQ+ diversity and inclusive care. Uncertainty regarding the recognition of partners and chosen families. Concerning the affordability and geographical accessibility of aged care services.	Fear of and distrust towards formal aged care services, leading to avoidance or delays in care planning. Increased anxiety about the future and a greater risk that support needs in later life will remain unmet.
Gillani B/2026 [40]	USA Constructivist grounded theory based on qualitative data from The Rainbow Connections Study, involving 28 LGBTQ+ participants of different ages, including older adults.	LGBTQ+ individuals (including younger and older adults, as well as transgender and non-binary people) and staff members from a community center who also provided caregiving and support services.	Discrimination, structural inequalities, and provider-level barriers that fragment individuals’ sense of self within healthcare systems.	Participants described four interrelated internal processes: integrating multiple aspects of the self, fostering intra-community support, engaging in self-determined care, and constructing personal meaning. Healthcare access extends beyond the utilization of services to encompass identity integration, community trust, and a sense of meaning. Accordingly, healthcare systems should provide care that promotes wholeness and actively addresses structural sources of harm.
Tissot D/2026 [41]	Australia Interpretive descriptive qualitative study using thematic analysis of LGBTIQA+ testimonies from the Royal Commission on Aged Care.	Care providers, informal caregivers, and community stakeholders involved in the care of LGBTIQA+ older adults.	Deep-seated fear of and distrust towards formal care services as a consequence of historical trauma. Inconsistent governance and a lack of accountability regarding diversity and inclusion standards. Absence of mandatory training and identity-affirming practices among the aged care workforce.	Engagement with and uptake of aged care services were severely undermined by fear and distrust. Genuine inclusion depends on operationalizing cultural safety through robust governance, clear accountability mechanisms, and adequately funded mandatory workforce training.
Johansen AV/2026 [21]	Integrative literature review (United States and other countries) examining the experiences of LGBT+ older adults in their interactions with healthcare systems across the life course.	LGB, transgender and transgender-diverse (TGD) older adults across a range of healthcare settings, including primary care, hospitals, and long-term care, as reported in the qualitative and quantitative studies included in the review.	Recurring themes included fear of disclosing one’s identity, previous experiences of discriminatory treatment, language and administrative forms that rendered sexual orientations and gender identities invisible, limited cultural competence among healthcare professionals, and a lack of dedicated LGBTQ+ resources.	These barriers resulted in avoidance or delays in seeking healthcare, perceptions of fragmented or incomplete care, and a range of unmet needs that adversely affected well-being and trust in the healthcare system. The review concluded that strengthening healthcare professionals’ cultural competence and expanding LGBTQ+-specific resources are essential.

**Table 3:** Level of evidence and grade of recommendation of the retrieved scientific literature.

Author	Year	LE *	GR
Silva JR et al. [25]	2021	2−	D
Kittle KR et al. [26]	2022	2+	C
Matthews AK et al. [27]	2022	3	D
Wang Y et al. [28]	2022	3	D
Gavulic K et al. [29]	2024	2+	C
Nguyen K et al. [30]	2024	2+	C
Franco-Rocha O et al. [31]	2024	2+	C
Perone A et al. [32]	2024	3	D
Sperling D. [33]	2024	3	D
Buckzak-Stec E et al. [34]	2025	2+	C
May J et al. [35]	2025	2+	C
Constantinou C et al. [20]	2025	2−	D
Prasad A et al. [36]	2025	2+	C
Adams A et al. [37]	2025	2+	C
Van Heesewijk J et al. [38]	2025	2+	C
Scott K et al. [39]	2025	2−	D
Gillani B et al. [40]	2026	3	D
Tissot D et al. [41]	2026	3	D
Johansen AV et al. [21]	2026	2−	D

* 2+ Well-conducted cohort or case–control studies with low risk of bias and with a moderate probability of establishing a causal relationship. 2− Cohort or case–control studies with high risk of bias and significant risk that the relationship is not causal. 3 Non-analytical studies, such as case reports and case series. This legend is taken from the Scottish Intercollegiate Guidelines Network (SIGN) guidelines [42].

## Data Availability

The data presented in this study are openly available in https://doi.org/10.17605/OSF.IO/52YJM (accessed on 8 July 2026).

## Supplementary Materials

The following supporting information can be downloaded at https://www.mdpi.com/article/10.3390/healthcare14193276/s1, Supplementary File S1. PRISMA-ScR checklist [23].
